# Gene expression changes in blastocyst hatching affect embryo implantation success in mice

**DOI:** 10.3389/fcell.2025.1496298

**Published:** 2025-02-06

**Authors:** Liyou An, Liang Zhang, Yulin Wu, Yadi Teng, Zihan Liu, Meixiang Ma, Miaolong Li, Xinrong Peng, Chenxi Liu

**Affiliations:** ^1^ Xinjiang Key Laboratory of Biological Resources and Genetic Engineering, College of Life Science and Technology, Xinjiang University, Urumqi, China; ^2^ Institute of Animal Biotechnology, Xinjiang Academy of Animal Science, Urumqi, China

**Keywords:** mouse blastocyst, hatching, gene expression, implantation, cell fate

## Abstract

In mammalian embryonic development, blastocyst hatching is essential for normal implantation and development of the fetus. We reported previously that blastocysts hatching out of the zona pellucida (ZP) exhibited site preferences that were associated with pregnancy outcomes. To characterize these site differences, we analyzed the transcriptomes in the following developing mouse blastocysts within 16 h of hatching: expanding (E), hatching from the A-site (A), B-site (B), and C-site (C), hatched (H), and non-hatching (N). By principal component analysis and hierarchical cluster analysis, we determined that the gene expression profiles of A and B blastocysts, which resulted in good fertility, clustered closely. C and N blastocysts, which resulted in poor fertility, clustered closely, but distantly from A and B. Embryos hatched at B- vs. C-sites, with good vs. poor pregnancy, showed 178 differentially expressed genes (DEGs), mainly involved in immunity, which correlated positively with birth rate. These DEGs were primarily regulated by transcription factors TCF24 and DLX3. During blastocyst hatching, immune-related genes were regulated, such as *Ptgs1, Lyz2, Il-α*, *Cfb* (upregulated) and *Cd36* (downregulated). By immunofluorescence staining, we found C3 and IL-1β on the extra-luminal surface of the trophectoderm of the hatched blastocyst, suggesting that they play a role in maternal-fetal interactions. As the blastocysts developed from the expanding to the fully hatched state, 307 DEGs were either upregulated by transcription factor ATOH8 or downregulated by SPIC to switch on immune pathways. Based on the hatching outcome, we identified three transcription patterns in developing blastocysts, with complex changes in the transcriptional regulation network of failed hatched blastocysts vs. successfully hatched blastocysts. We developed a LASSO regression-based model using DEGs *Lyz2, Cd36, Cfb*, and *Cyp17a1* to predict implantation success. This study revealed the diverse, multidimensional developmental fates of blastocysts during short-term hatching and indicated that the immune properties of the embryo had a major effect on blastocyst hatching outcomes. We suggest that transcriptional changes and their regulation during the development of the preimplantation blastocyst affect implantation. This study contributes to our understanding of the role of transcriptional changes in mammalian embryonic development during hatching and their effect on maternal-fetal interactions.

## 1 Introduction

Mammalian embryonic development begins with fertilization, which is followed by cell proliferation and differentiation, forming the blastocyst that is surrounded by the trophectoderm (TE) and the inner cell mass (ICM). As the blastocyst cavity expands, the embryo hatches from the zona pellucida (ZP), directly contacts the uterine endometrium, initiating embryo implantation and pregnancy, and develops into a fetus (Terakawa et al., 2012; Ma et al., 2024). Blastocyst hatching is a consequence of elevated osmotic pressure due to active Na^+^/K^+^ ion transporters in TE cells and proteases produced by TE that hydrolyze the ZP (Leonavicius et al., 2018). Blastocyst hatching is an essential preimplantation event that promotes physiological/molecular embryo-uterine crosstalk to initiate embryo implantation. During hatching, there are changes in the blastocyst phenotype, including where the TE initially hatches out of the ZP, the outcome of hatching, and the molecules involved in hatching (Liu et al., 2022). In blastocyst hatching, steroid hormones, growth factors, cytokines, enzymes, and transcription factors coordinately regulate the development of preimplantation embryos and create a favorable environment for embryo implantation (Leonavicius et al., 2018; Wang and Dey, 2006). Successful hatching and implantation involve specific spatiotemporal patterns and dynamic expression of many factors (Ma et al., 2024; Ma et al., 2019); however, the process is not well understood. As previous studies have focused on blastocyst cell-lineage differentiation and regulation of blastocysts and post-implantation development (Leonavicius et al., 2018; Wang et al., 2023; Arutyunyan et al., 2023), it is unclear how the fate of preimplantation embryos is determined and how it affects embryo implantation. In assisted reproductive technology (ART) practice, we cannot predict whether a blastocyst will develop normally after transfer and implant successfully (Rienzi et al., 2017; Knudtson et al., 2017).

There are few studies on the role of blastocyst hatching in implantation. Liu et al. reported a preference for certain hatching sites in mouse blastocysts (Liu et al., 2020), and we also demonstrated site preferences for blastocyst hatching, which determine the efficiency of implantation and pregnancy outcomes (An et al., 2021). With the ICM at the 12 o’clock position, blastocyst hatching is classified into five patterns: O-site (12 o’clock), A-site (1–2 o’clock), B-site (3 o’clock), C-site (4–5 o’clock), and D-site (6 o’clock). We found that 81.8% of blastocysts hatched from near or beside the ICM (A-site and B-site, respectively), whereas 15.6% hatched opposite the ICM (C-site and D-site). After embryo transfer, the birth rate was highest for blastocysts that hatched from the B-site (65.6%), higher than for the C-site (21.3%) or the control group (expanding blastocysts, 41.3%), with no significant difference compared to the A-site (55.6%). The failure of blastocyst hatching results in a birth rate of only 5.1% following embryo transfer (An et al., 2021). In ART, the ZP usually hardens as a result of *in vitro* culture, embryo cryopreservation, or artificial embryo manipulations (Rienzi et al., 2017). Assisted hatching techniques are commonly used in clinical ART To facilitate embryo hatching, but their efficacy is controversial (Knudtson et al., 2017; Kissin et al., 2014). Blastocyst hatching shows site preference, and embryos hatched from the vicinity of the ICM have a higher implantation efficiency and birth rate; therefore, we developed a modified assisted hatching technique that treated the ZP at the specified site, resulting in a birth rate of 77.1% for treated B-site blastocysts after embryo transfer and confirmed the importance of the hatching site on implantation (An et al., 2021).

Although blastocyst hatching affects embryo implantation, the molecular aspects of development in blastocyst hatching are poorly understood (Liu et al., 2022; Ilina et al., 2021). In this study, we performed a multi-level molecular analysis incorporating RNA-seq, RT-qPCR and immunofluorescence to characterize transcriptional changes during embryo hatching. Additionally, we aimed to explore the differential development of hatching blastocysts that may influence hatching site preferences, hatching outcomes and implantation. Furthermore, we developed a predictive model for blastocyst pregnancy outcomes. This study provides new insights into blastocyst development and cell fate that may lead to new methods to optimize ART.

## 2 Materials and methods

### 2.1 Animal care and ethical approval

All animal care and use procedures were approved by the Animal Care and Use Committee of Xinjiang University (IACUC-20210709) and performed according to the guidelines of the U.S. National Institutes of Health. Female CD-1 mice (6–8 weeks old) and male CD-1 mice (8–9 weeks old) were purchased from the Animal Resource Centre of Xinjiang Medical University and housed in a specific pathogen-free facility of Xinjiang University under a standard 12-h light/12-h dark cycle with free access to food and water.

### 2.2 Reagents

Reverse transcription reagents and qPCR reagents were purchased from Applied Biological Materials Inc (abm, Richmond, Canada). Unless otherwise specified, other reagents were purchased from Sigma-Aldrich (MO, United States).

### 2.3 Mouse treatments and embryo collection

**FIGURE 1:**
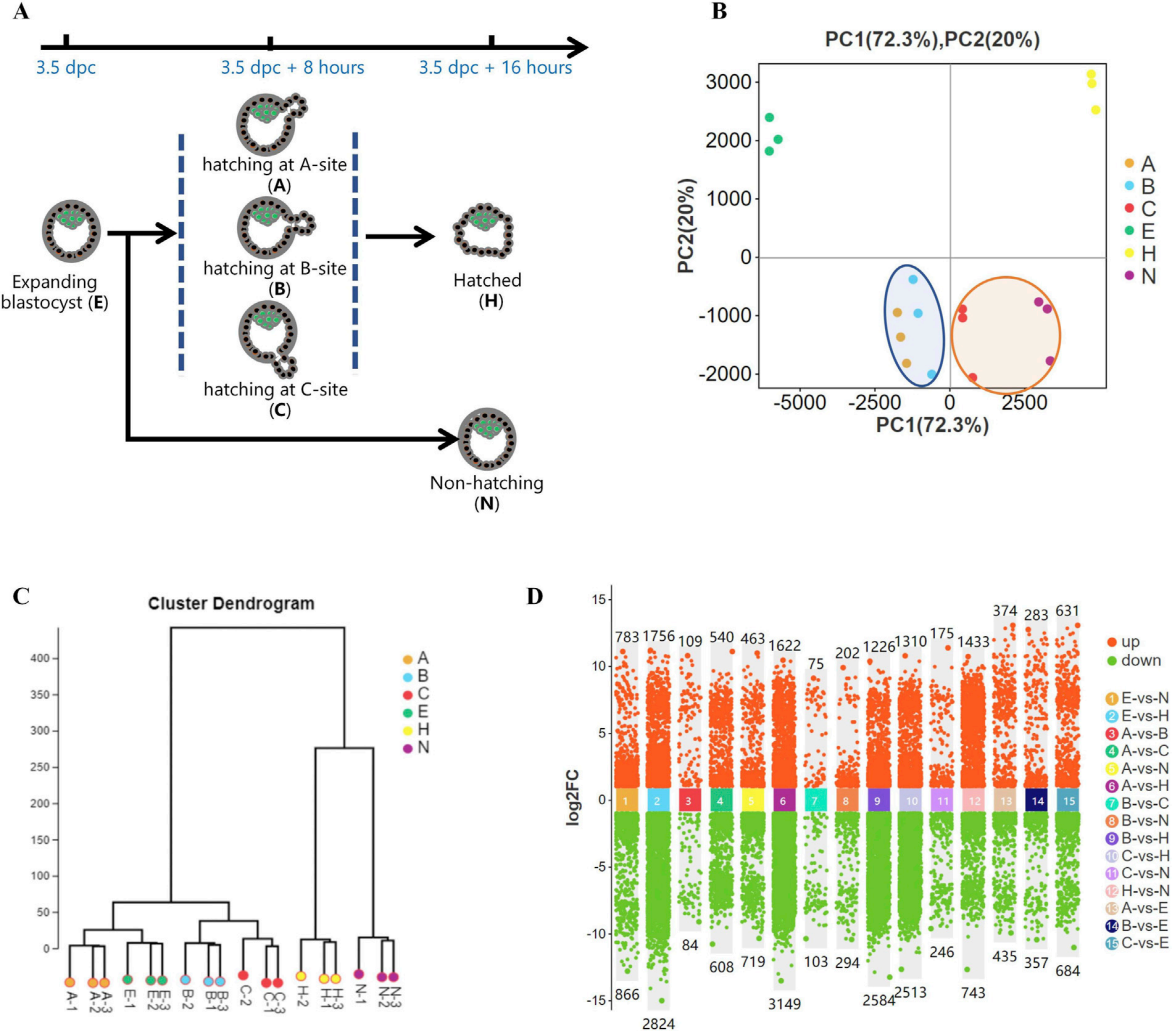
Gene expression differences during blastocyst hatching and development. **(A)** Most expanding blastocysts (3.5 dpc) developed to the hatching state at about 8 h (3.5 dpc +8 h) and hatched at about 16 h (3.5 dpc +16 h). The remaining blastocysts failed to hatch (3.5 dpc +16 h). Blastocysts were classified into five groups according to their hatching states, including expanding (E), hatching at A-site (A), hatching at B-site (B), hatching at C-site (C), hatched (H), and non-hatching/hatching failure (N) and analyzed by Smart-Seq. **(B)** Global gene expression was analyzed by principal component analysis (PCA). It showed that the gene expression profiles of A and B blastocysts, which resulted in good fertility, clustered closely (circled in blue). C and N blastocysts, which resulted in poor fertility, clustered closely (circled in yellow). **(C)** Hierarchical cluster analysis revealed two main clusters of gene expression in developing blastocysts. One cluster comprised expanding blastocysts and those hatching at A, B, and C, and the other comprised blastocysts hatched and non-hatched blastocysts. **(D)** Comparison of differentially expressed genes (DEGs) for each blastocyst group.

Female mice were treated with pregnant mare serum gonadotropin (PMSG) and human chorionic gonadotropin (high) to induce superovulation and were mated with male mice as previously described (Wu et al., 2013). With the observation of a copulatory plug the next morning, the pregnancy stage was considered 0.5 days post-coitus (dpc). The uterus with a short Fallopian tube was recovered at 3.5 dpc. Subsequently, a blunt 30-gauge needle was inserted into the Fallopian tube, and expanding blastocysts were flushed from the uterus using 200 μL of M2 medium. Embryos were collected and cultured in KSOM medium under mineral oil. After culture for 6–8 h, blastocyst hatching was classified based on the hatching site (A-site, B-site, or C-site) (An et al., 2021). After 16 h of culture, blastocysts were divided into hatched and hatching failure groups (hatching embryos, H; non-hatching embryos, N; Figure 1A).

### 2.4 RNA sequencing (Smart-Seq)

Total RNA extracted from embryos using TRIzol method. Expanding blastocysts (E), blastocysts with A-site, B-site, or C-site hatching sites (A, B, C), hatched blastocysts (H), and non-hatching embryos (N) were analyzed by RNA sequencing (Figure 1A). In each group, a panel of 30 embryos was collected and stored in 100 μL of TRIzol (Thermo Fisher Scientific, MA, United States) with three replicates (total 90 embryos). Transcriptome sequencing with Smart-Seq (Goetz and Trimarchi, 2012) was conducted by the Guangzhou GENE DENOVO Company. Differentially expressed genes (DEGs) were analyzed by EdgeR and normalized in fragments per kilobase of transcript per million mapped reads (FPKM, see Supplementary Table 1). DEGs and gene sets were analyzed using Gene Ontology (GO) term enrichment and Kyoto Encyclopedia of Genes and Genomes (KEGG) analysis using the online analysis system (Guangzhou GENE DENOVO Company, Guangzhou, China). In transcription factor-target gene regulatory network analysis, transcription factors (TFs) were identified in the selected gene set, the JASPAR database was used to obtain transcription factor binding motifs, and MEME FIMO software was used to predict the transcription target genes in the selected set. A transcription factor targeting network showed the association between TFs and target genes.

### 2.5 Single-blastocyst reverse transcriptase-qPCR

A pool of 10 embryos in each group was collected, and cDNA was synthesized by All-In-One 5 × RT MasterMix according to the manufacturer’s instructions (abm). For single embryo measurements, we lysed one embryo for cDNA synthesis using a scaled-down reaction based on the manufacturer’s instructions (abm). Simply, cDNA synthesis was modified by treating an embryo in a micro-drop (5 μL) scaled-down reaction solution on a plastic dish covered with mineral oil to prevent evaporation of fluids. The cDNA was stored at −20°C for qPCR. Through transcriptome analysis we identified ten key genes (*Cd36*, *Ccl9*, *C3*, *Cyp17al*, *Il-1a*, *Ccl5*, *Susd4*, *Cfb*, *Ptgs1*, and *Lyz2*) for analysis and designed qPCR primers for them (primer sequence see Supplementary Table 2). Those 10 key genes selected from GO and KEGG enrichment in immunity and with relatively high expression (FPKM>1). The housekeeping gene *Gapdh* was used for RNA normalization. The 20-μL qPCR reaction comprised 10 μL of Blasaq 2 × qPCR Master Mix (abm), 0.5 μL of each upstream and downstream primer, and 2 μL of cDNA. The products were electrophoresed on a 2% agarose gel to confirm amplification. For RT-qPCR analysis, data were obtained from three technical replicates in each experiment and three biological replicates in each blastocyst group.

### 2.6 Immunofluorescence analysis

Expression of the protein products of *C3, Cdx2, Il-1β, Lyz2,* and *Plac1* was determined using immunofluorescence (IF) as previously described (An et al., 2021). Briefly, the embryos were washed three times in 0.1% polyvinyl alcohol (PVA) for 3–5 min, fixed in 4% paraformaldehyde buffer for 20 min, followed by three washes with 0.1% PVA. Embryos were permeabilized with 0.5% Triton X-100 at room temperature for 15 min, washed three times with 0.1% PVA, and blocked in 4% fetal bovine serum at room temperature for 1 h. Embryos were incubated with the primary antibody C3, Cdx2, IL-1β, Lyz2 and Plac1 respectively (diluted 1:200 in 2% phosphate-buffered saline, Abcam, Cambridge, United Kingdom), at 37°C for 1 h or at 4°C overnight. Embryos were washed with 0.1% PVA and incubated in the corresponding secondary antibody (goat anti-rabbit IgG AF488, Abcam) in the dark at 37°C for 1 h, washed three times with 0.1% PVA, and stained with 4′,6-diamidino-2-phenylindole (DAPI) for 5 min. Images were acquired using a laser-scanning confocal microscope (Nikon, Japan).

### 2.7 Development of a prediction model by least absolute shrinkage and selection operator regression analysis

Using the R language survival and glmnet package, we prepared the input data and then built the LASSO regression model. We selected the appropriate λ value and remodeled the data using this λ value. We performed cross-validation on the LASSO analysis results to determine the gene data to be included in the model, ensuring a rational and effective model.

### 2.8 Experimental design

Blastocysts were classified into five groups according to their hatching status, including expanding blastocysts (E), hatching blastocysts with A-site (1-2 o’clock), B-site (3 o’clock), C-site (4-5 o’clock) hatching sites (A, B, C), hatched blastocysts (H) and non-hatching embryos (N). Blastocysts hatching at O-site (12 o’clock) and D-site (6 o’clock) were excluded due to the rarity of hatching events and the difficulty of collecting enough embryos for analysis. First, gene expression profiles were analyzed to describe the general state of development during blastocyst hatching. Then, to explain how blastocyst hatching determines its implantation, a comparative analysis was performed in blastocysts hatching from the B-site (good pregnancy outcome) vs. the C-site (poor pregnancy outcome). To reveal how hatching occurs and is achieved, blastocysts were analyzed at the stages from expansion to hatching and hatched. To reveal the intrinsic determinants of different hatching outcomes, hatched and non-hatched blastocysts were analyzed comparatively. Based on the above data analysis, a predictive model of implantation success was developed. A modified single blastocyst gene expression detection approach was established to confirm the expression profiles of key genes in the prediction model.

### 2.9 Statistical analysis

The data obtained for RT-qPCR and IF are presented as mean ± standard error of the mean (SEM). One-way analysis of variance with a *post hoc* two-sided Sidak *t*-test was used to determine differences between treatment groups. All statistical analyses were performed using GraphPad Prism 9.01 for Windows, version 24.0 software (GraphPad Software, La Jolla, CA, United States). A *p* < 0.05 difference between groups was considered significant.

## 3 Results

### 3.1 Gene expression profiles during blastocyst hatching and development

We previously found a strong association between the birth rate and the hatching states in mice (An et al., 2021). To further investigate this, we performed Smart-Seq on pre-hatching or expanding blastocysts at 3.5 dpc (E); blastocysts hatching from A-site (A), B-site (B), and C-site (C) at 3.5 dpc plus 8 h; and hatched blastocysts (H) as well as non-hatching blastocysts (N) at 3.5 dpc plus 16 h (Figure 1A),consistent with research groups as previous study (An et al., 2021). Global gene expression, analyzed by principal component analysis (PCA), showed distinct groups based on hatching states (Figure 1B). A and B, which resulted in higher embryo implantation (55.6%, 65.6%) (An et al., 2021), clustered closely at the left side of the plot, whereas C and N, with very low birth rates of 21% and 5.2%, respectively, clustered closely. In addition, fully hatched blastocysts formed a distinct cluster. Hierarchical cluster analysis of gene expression segregated the developing blastocysts into a cluster that comprised expanding blastocysts and hatching A, B and C blastocysts. The other cluster comprised H and N blastocysts (Figure 1C). Thus, blastocysts with different pregnancy outcomes had marked differences in gene expression profiles. Moreover, as a blastocyst developed from expanding to hatching to hatched, its gene expression profile changed dramatically (Figure 1D), reflecting blastocyst development during this process.

### 3.2 Differences in gene expression during blastocyst hatching and development affect pregnancy success

**FIGURE 2:**
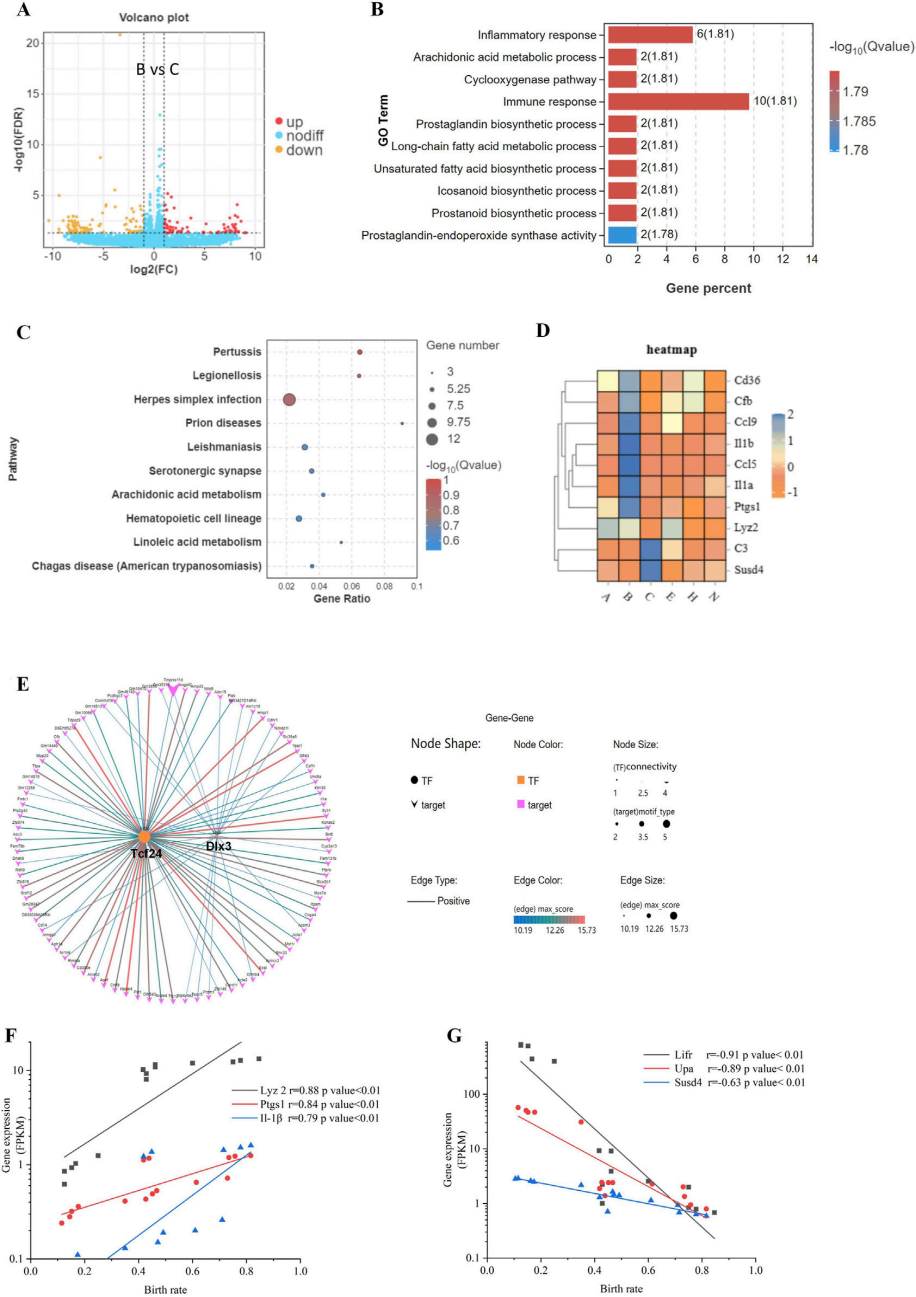
Differences in gene expression during blastocyst hatching and development that affect pregnancy success. **(A)** The differential gene expression between B and C blastocysts, which had the greatest difference in birth rates (65.6% vs. 21.3%), was analyzed and visualized as a volcano map. **(B)** DEGs were analyzed by Gene Ontology (GO) functional enrichment for the top 10 significant biological events. **(C)** The top 10 GO signaling pathways were enriched by the Kyoto Encyclopedia of Genes and Genomes (KEGG) analysis. **(D)** Expression patterns of immune-related genes, enriched in GO and KEGG, visualized as a heatmap for each blastocyst state. **(E)** Transcription factor-target gene regulatory network analysis of DEGs identified enriched TFs as *Tcf24* and *Dlx3.* The expression of three upregulated DEGs **(F)** and three downregulated DEGs **(G)** was analyzed for correlation with the birth rate.

Comparing the blastocysts hatching from the B-site (65.6% birth rate) vs. the C-site (21.3% birth rate) (An et al., 2021), we found that 75 genes were upregulated, and 103 genes were downregulated (Figure 2A). Among DEGs, 1) biology process of inflammatory response and immune response, and 2) pathway of infectious disease were highly enriched by GO and KEGG analysis (Figures 2B, C). As immune genes *Cd36, Cfb, Ccl9, Il-1β, Ccl5, Il-1α, Ptgs1, Lyz2, C3,* and *Susd4* showed significant differences between B-site and C-site blastocysts (Figure 2D), immunological changes in the hatching blastocyst likely allow it to recognize the endometrium at embryo implantation.

For B-site and C-site hatching blastocysts, DEGs fell into groups with > 5-fold change or < 5-fold change (Figure 2A), suggesting that genes with a > 5-fold change may be transcriptionally coregulated during the 8 h of blastocyst hatching. Indeed, we found by transcription factor-target gene regulatory network analysis that 2 TFs, TCF24 and DLX3, controlled over 82 out of 178 DEGs (46%) (Figure 2E). We selected four DEGs, *Lyz2*, *Ptgs1*, *Il-1β, and Susd4*, and two previously investigated genes significantly correlating with implantation, *Lifr* and *Upa* (An et al., 2021), and analyzed the correlation of their expression level with birth rate. *Lyz2*, *Ptgs1*, and *Il-1β* were positively correlated with birth rate, whereas *Lifr*, *Upa*, and *Susd4* were negatively correlated with birth rate (Figures 2F, G). Differentiation genes coordinate and determine developmental fate during blastocyst hatching. Therefore, these gene products may be involved in maternal-fetal interactions during implantation, which helps to determine pregnancy outcomes.

### 3.3 mRNA and protein expression of key genes in embryo hatching

**FIGURE 3:**
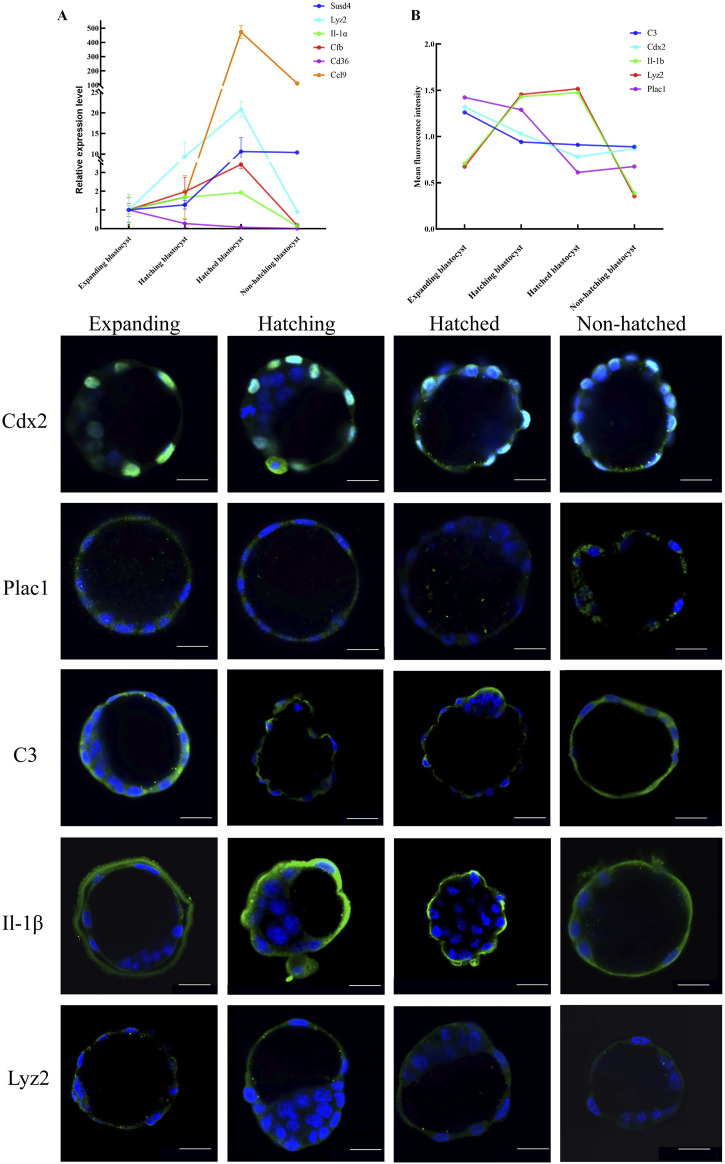
Expression patterns of key genes in embryo hatching. **(A)** Relative expression of the immune-related genes enriched in KEGG was measured by RT-qPCR expressed as the fold change determined by 2^−ΔΔCT^ normalized the housekeeping gene *Gapdh*. **(B)** Protein expression for CDX2, PLAC1, C3, IL-1β, and LYZ2 was determined by green IF staining in embryos imaged by confocal microscopy during the blastocyst hatching process expressed as relative mean fluorescence intensity. All nuclei were labeled with DAPI (blue). Scale bar, 20 μm.

We used RT-qPCR to measure transcription of immune-related genes *Ptgs1*, *Lyz2*, *Il-α*, *Cfb*, *Ccl9, Cd36*, *Ccl5*, *C3*, *Cyp17 al*, and *Susd4* in five blastocysts each from expanding, hatching, hatched, and non-hatching groups. We found upregulation of *Lyz2*, *Il-α*, *Cfb, Ccl9* and *Susd4,* and downregulation of *Cd36* expression during blastocyst hatching (Figure 3A). *Ccl5*, *C3, Cyp17al*, and *Ptgs1* exhibited low expression levels or were undetectable. There was a different level of gene expression in the non-hatching blastocyst. The RT-qPCR gene expression patterns were consistent with the transcriptome analysis.

We used immunofluorescence (IF) microscopy to measure genes Cdx2 and Plac1 (regulating trophoblast differentiation), C3, IL-1β, and Lyz2 (immune-relative genes) in each hatching state (Figure 3). IF signals for Plac1, C3, IL-1β, and Lyz2 were mainly in the cytoplasm of trophoblast cells, whereas Cdx2 was expressed in the nuclei of trophoblast cells (Figure 3). Notably, C3 and IL-1β were expressed on the extra-luminal surface of the TE of the hatched blastocysts, implying that these proteins play a role in maternal-fetal interactions when the embryo comes into contact with the endometrium. However, in non-hatched blastocysts, this protein translocation was not detected. Based on mean fluorescence intensity, we found a decrease in the expression of Plac1*,* Cdx2, and C3 and an increase in the expression of Lyz2 and IL-1β from expanding to hatching blastocysts (Figure 3B) consistent with the transcriptomic and RT-qPCR results. Non-hatching blastocysts had lower levels of Lyz2 and IL-1β compared to hatched blastocysts, showing a dysregulation of these proteins.

### 3.4 Differential gene expression and associated processes that regulate development during blastocyst hatching

**FIGURE 4:**
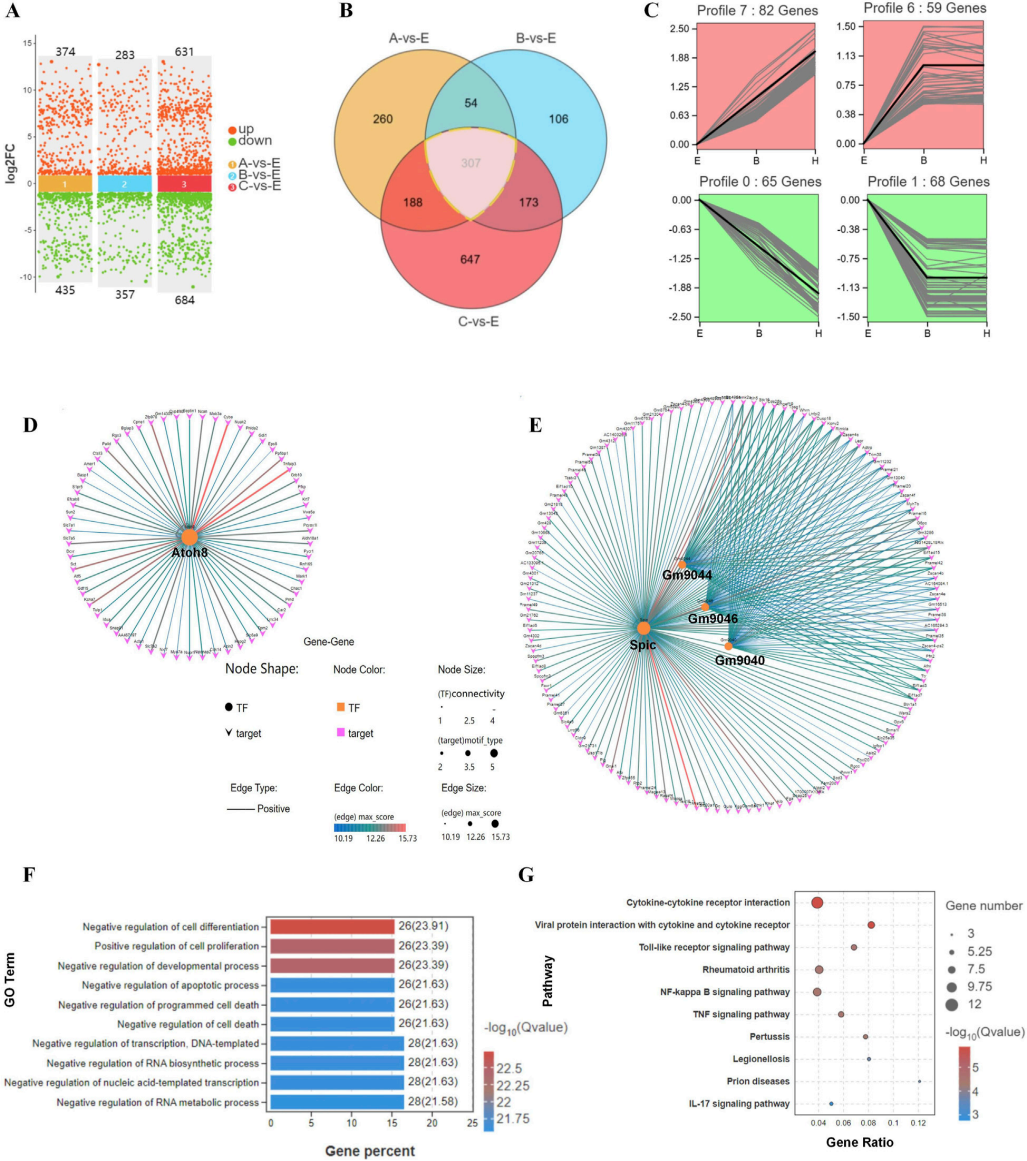
Differential gene expression and their associated processes regulate differentiation during blastocyst hatching. **(A)** DEGs were compared in E, A, B, and C blastocysts. **(B)** DEGs were analyzed by Venn diagram, and 307 genes were common to all comparisons of blastocyst hatching (circled by a dotted line). **(C)** Trend analysis identified genes with various types of expression profile changes from blastocyst expanding to hatching and hatched. These genes were analyzed by transcription factor-target gene regulatory network analysis, which identified TFs ATOH8 in profiles 6 and 7 **(D)**, and SPIC, GM9044, GM9046 and GM9040 in profiles 0 and 1 **(E)**. The genes identified by trend analysis were analyzed by GO functional enrichment for the top 10 significant biological processes **(F)** and KEGG pathway enrichment for the top 10 significant signaling pathways **(G)**.

We analyzed the DEG expression patterns during the hatching process for E, B, and C blastocysts by Venn diagrams (Figures 4A, B) and found that the expression of 307 genes correlated with blastocyst hatching, thereby determining blastocyst development from expanding to hatching (A-site, B-site, and C-site). By trend analysis, the 307 DEGs showed four significant gene expression patterns, with 141 genes in profiles 6 and 7 showing an upregulated trend and 133 genes in profiles 0 and 1 showing a downregulated trend (Figure 4C). Thus, there were two dominant sets of DEGs that were associated with blastocyst development during hatching. By transcription factor-target gene regulatory network analysis, we found that 1) TF ATOH8 regulated 41.8% of DEGs with an upregulated trend (59/141, Figure 4D), 2) TFs SPIC, GM9044, GM9046 and GM9040 regulated 97% of DEGs with a downregulated trend (129/133, Figure 4E), and 3) SPIC was a major regulator of coregulated *Gm9044, Gm9046 and Gm9040*.

By GO analysis of DEGs, the top 10 enriched terms included the regulation of cell differentiation, cell proliferation, and developmental and apoptotic processes (Figure 4F). In the KEGG analysis, the top 10 enriched signaling pathways included cytokine-cytokine receptor interaction, viral infection, and a Toll-like receptor signaling pathway (Figure 4G). Thus, remodeling of cell processes and the shaping of immunological properties were the major developmental events during blastocyst hatching.

### 3.5 Biological processes associated with changes in transcriptional profiles during development of blastocysts with various hatching outcomes

**FIGURE 5:**
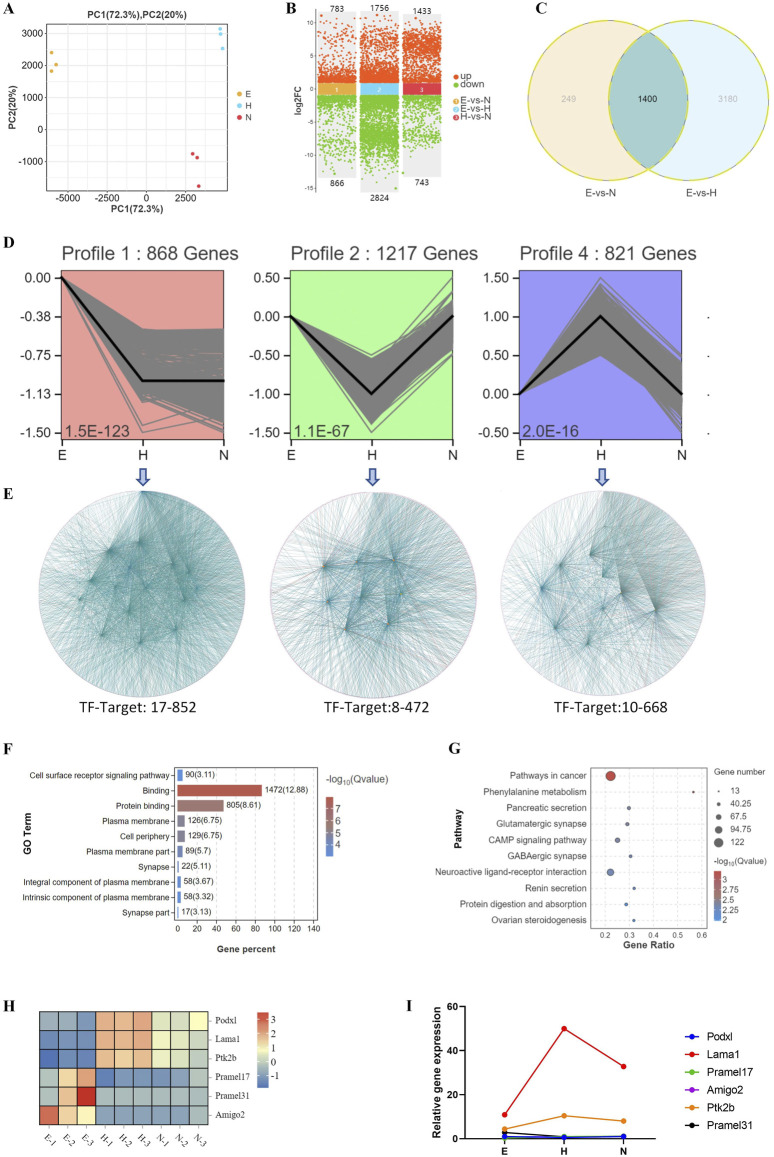
Biological processes associated with changes in transcriptional profiles during development of blastocysts with various hatching outcomes. **(A)** DEGs were analyzed by PCA in blastocysts with different hatching outcomes. **(B)** DEGs were compared in hatching blastocysts and non-hatched blastocysts. **(C)** DEGs were analyzed by Venn diagram among E (expanding), H (hatched), and N (non-hatched) blastocyst; 3,428 genes correlated with different hatching outcomes (circled by dotted line). **(D)** Trend analysis identified genes with three types of expression profile changes for blastocysts with different hatching outcomes. **(E)** The genes identified by trend analysis were further analyzed by transcription factor-target gene regulatory network analysis. The genes identified by trend analysis were analyzed by GO functional enrichment for the top 10 significant biological processes **(F)** and by KEGG pathway enrichment for the top 10 significant signaling pathways **(G)**. **(H)** The expression patterns of selected genes identified by trend analysis are shown in a heatmap. **(I)** Transcription of these genes was confirmed by RT-qPCR. The relative expression level shown as fold changes was calculated by 2^−ΔΔCT^, normalized to the housekeeping gene *Gapdh*.

PCA analysis of DEGs for E, H, and N blastocysts revealed clear clusters for E, H, and N blastocysts, showing a low correlation between hatched and non-hatched blastocysts (Figure 5A). In non-hatched blastocysts, 1,433 genes were upregulated, and 743 genes were downregulated compared with hatched blastocysts (Figure 5B). Venn diagram analysis of DEGs for E, N, and H blastocysts showed that 3,428 genes correlated with differential hatching outcomes (Figure 5C), thereby determining whether blastocysts hatched. By trend analysis, we determined that 1) 868 genes showed a decreasing trend, 2) 1,217 genes showed a V-shape trend, and 3) 821 genes showed an inverted V-shaped trend (Figure 5D). For these three trends, transcription factor-target gene regulatory network analysis identified 17 TFs (852 target genes), 8 TFs (472 target genes) and 10 TFs (668 target genes) (Figure 5E), indicating complex patterns of transcriptional gene regulation that determine whether the blastocyst will hatch. Changes in the expression of these genes, which were enriched in processes related to cell surface receptor signaling, binding, and the plasma membrane (Figure 5F), affect the hatching outcome. KEGG analysis showed that the top enriched signaling pathways were related to cancer, phenylalanine metabolism, and the cAMP signaling pathway (Figure 5G). These GO and KEGG results demonstrate that during hatching, these molecular events and their transcriptional regulation control the processes that lead to blastocyst development.

We used RT-qPCR to measure the transcription of genes associated with these trends, including *Podxl*, *Lama1*, *Ptk2b*, *Pramel17*, *Pramel31*, and *Amigo2*. *Lama1* and *Ptk2b* were upregulated, whereas expression of *Pramel17*, *Amigo2, Pramel31*, and *Podxl* was low or undetectable in blastocyst hatching (Figure 5I), consistent with their transcriptomic expression patterns (Figure 5H).

### 3.6 A model to predict implantation success for hatching blastocysts

**FIGURE 6:**
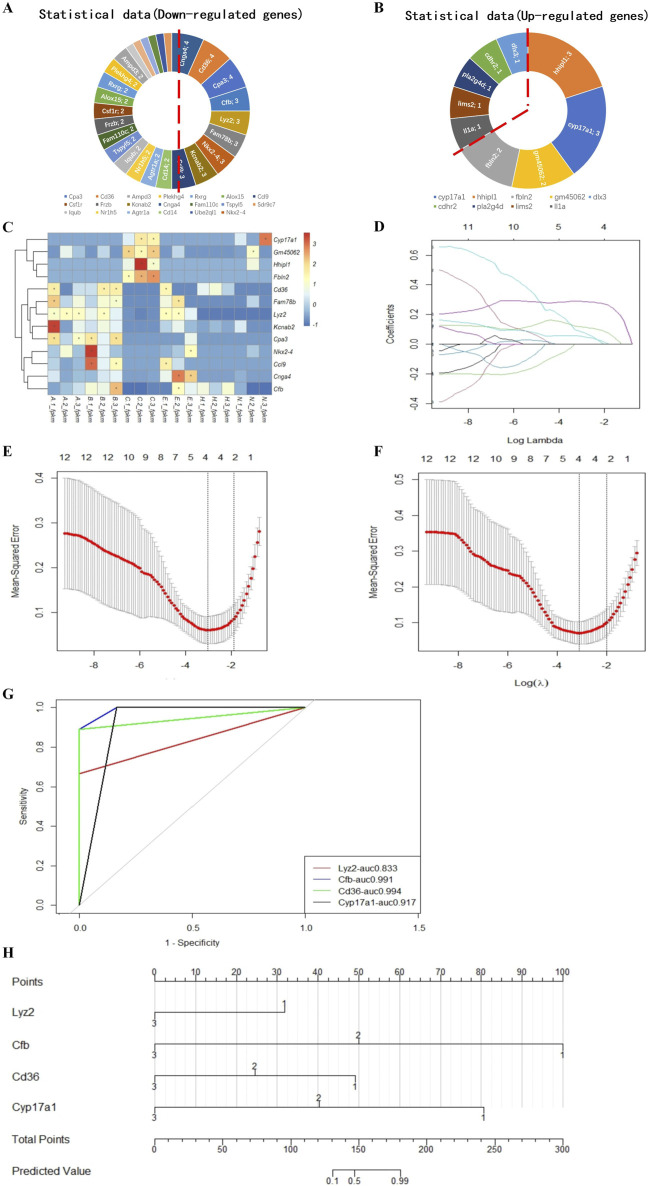
Model for predicting the implantation success for hatching blastocysts. Nine downregulated candidate genes from the expression trends **(A)** and four upregulated genes **(B)** are shown in a heatmap **(C)**. These genes were analyzed using the LASSO regression model. **(D)** Each curve represents a coefficient, and the *x*-axis represents the regularization penalty parameter. As the tuning parameter (λ) changes, a coefficient that becomes non-zero enters the LASSO regression model. **(E, F)** Cross-validation to select the optimal λ. The red dotted vertical line crosses over the optimal log λ, which corresponds to the minimum value for multivariate Cox modeling. The two dotted lines represent one standard deviation from the minimum value. **(G)** The predictive performance of the model was evaluated by receiver operating characteristic curve analysis. The evaluated variables (genes) were used to construct an implantation potential prediction model by nomogram graph **(H)**. In this predictive model, gene expression levels corresponded to a score. The scores for each gene were added to obtain the total points, which corresponded to the predicted values that specify the risk/potential for implantation outcomes.

We ranked the DEGs associated with blastocyst development from the transcriptome analysis and identified 13 candidate genes to develop a model to predict blastocyst implantation success. The nine DEGs that were downregulated in three out of four measurements and the four DEGs that were upregulated in two out of three measurements (Figures 6A, B) are shown in a heatmap (Figure 6C). Predictive aggregate effects were identified using LASSO regression (Figure 6D). To determine the gene variables to include in the model and to ensure a rational, effective model, we used LASSO regression analysis using the R language survival and glmnet package, followed by cross-validation of the LASSO analysis results (Figures 6D–F). We screened *Lyz2*, *Cd36*, *Cfb*, and *Cyp17a1* as variables using LASSO regression and determined by LASSO Cox regression analysis that these four genes, the minimum value for multivariate Cox modeling, were statistically significant. To determine the predictive performance of the model, we evaluated these genes using receiver operating characteristic (ROC) curve analysis, which showed that they made a significant contribution to the prediction of the model, with area under the ROC curve values generally greater than 0.8 (Figure 6G).

Using these validated genes as the variables, we plotted a nomogram graph based on the LASSO regression and cross-validation data (Figure 6H) to produce a predictive model of implantation success for hatching blastocysts. In this predictive model, the level of expression corresponded to the score for each gene. For example, if the relative expression level of *Lyz2* was 1, this gene was given a score of 32. The scores for each gene were added together to produce a total score, which corresponded to the predicted value, which indicated the potential implantation outcome for a blastocyst.

### 3.7 Expression of genes used in the prediction model

**FIGURE 7:**
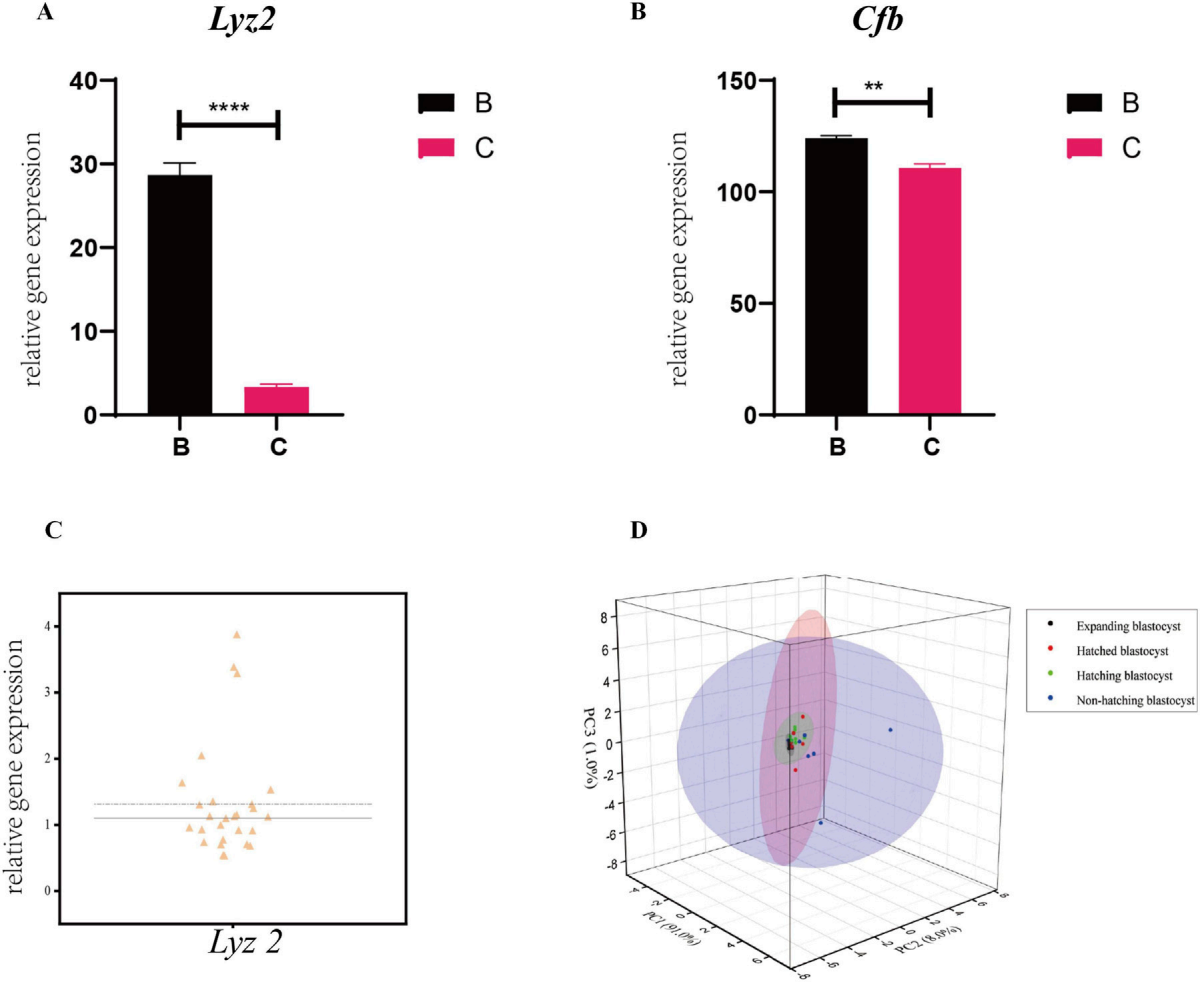
Expression of the genes in the prediction model in single blastocysts. Expression of *Lyz2* **(A)** and *Cfb* **(B)** was measured by RT-qPCR in single blastocysts hatching at B- and C-sites. All data are represented as mean ± SEM (n = 3). Asterisk denotes statistically significant differences *p < 0.05; **p < 0.01; ***p < 0.001; ****p < 0.0001. **(C)** Variability in the expression of *Lyz2* was observed in different populations of pre-hatching/expanding blastocysts. **(D)** *Lyz2* expression profiles in blastocyst populations at the expanding, hatching, hatched, and non-hatched stages were analyzed by PCA.

We measured the expression of the genes in the prediction model in single blastocysts by RT-qPCR. For a single blastocyst hatching at the B- and C-sites, we found that the expression of *Lyz2* and *Cfb* was consistent with data obtained from pooled blastocyst RT-qPCR, confirming the validity of the single-blastocyst technique (Figures 7A, B). Differences in the expression of *Lyz2* were observed across 27 populations of expanding blastocysts, suggesting that the expression of *Lyz2* is regulated by the intrinsic programming fate before hatching (Figure 7C). PCA analysis of *Lyz2* expression profiles in blastocyst populations from the expanding, hatching, hatched, and non-hatched stages showed strong clustering for blastocysts in the expanding state, weaker clustering for blastocysts at the hatching state, and poor clustering for cells at the hatched stage (Figure 7D). However, based on *Lyz2* expression, blastocysts that failed to hatch were dispersed (Figure 7D).

## 4 Discussion

We found previously that the hatching site affects blastocyst implantation and a successful pregnancy (An et al., 2021). In this study, we determined the role of changes in gene expression in blastocyst development during the short-term hatching process to identify the key proteins and pathways that influence implantation outcomes.

Embryo implantation, which requires close contact between a developmentally competent blastocyst and a receptive uterus, includes blastocyst localization, adhesion and invasion, as well as placenta formation (Wang and Dey, 2006; Matsumoto, 2017). Blastocyst hatching, which is the emergence of the embryo from the ZP, is critical for successful implantation in mammalian embryos (Leonavicius et al., 2018). Any factors adversely affecting blastocyst hatching can lead to implantation failure (Zhan et al., 2018). Hatching plays a critical role in the interaction between the embryo and the maternal environment, determining whether the embryo implants, producing a successful fetus (Wang et al., 2023; Cheng et al., 2023). The relationship between embryonic development during hatching and maternal recognition in mammals is unknown. Previously, we reported site preferences in blastocyst hatching, which determines the pregnancy outcome of embryos (An et al., 2021). In this study, we found that blastocysts that differ in their various pregnancy outcomes had marked differences in gene expression profiles, indicating differences in blastocyst development. The DEGs between blastocysts hatched at the B- and C-sites were mainly related to immune function. We found that most DEGs were regulated by common TFs, suggesting that the implantation fate of blastocysts is regulated during hatching. Implantation requires dynamic bidirectional communication between the blastocyst and the uterine endometrium (Jones-Paris et al., 2017). Maternal immune cells recognize embryonic signals, inducing an immune tolerance that aids in embryo implantation (Fujiwara et al., 2016). However, the immune signals produced by the embryo during hatching that induce the uterine response are poorly understood.

In embryonic development, heterogeneity of cell division is important in determining cell fate at the 2-cell stage, whereas cell position is critical for lineage differentiation at the 8-cell stage. In the morula stage, multiple embryonic layers are differentiated, followed by germ layer differentiation at the blastocyst stage (Zhang et al., 2013; Hernandez Mora et al., 2023; Dang et al., 2023). Previous studies on cell fate have not found a correlation between early embryo differentiation and implantation potential (Clyde, 2022). However, blastocyst hatching is a critical juncture in the transition from early embryo development to implantation (Tvergaard et al., 2021). We found that blastocyst hatching determines implantation and pregnancy outcomes (An et al., 2021), suggesting that within the short hatching period, changes in embryonic development lead to different implantation fates. Here, we showed that gene expression patterns in blastocysts as they proceeded from expanding to hatching were characterized by upregulated and downregulated DEGs regulated by TFs. The DEGs were mainly related to immunity and inflammation, such as *Ptgs1*, *Lyz2*, *Il-α*, *Cfb*, *Ccl9*, *Cd36*, *Ccl5*, and *C3*. Chemokine genes *Ccl9* and *Ccl5* were highly expressed in hatching blastocysts. The *Ccl5* chemokine receptor CCR1 induces myeloid-derived suppressor cell recruitment (He et al., 2016). The balance between proinflammatory factors and the anti-inflammatory cytokine CCL9 ensures effective embryo-uterus recognition with a tolerable immune response (Robertson et al., 2018). There are many studies of the immunological processes during embryo implantation; however, specific cellular and molecular interactions are poorly characterized (Oghbaei et al., 2022). We found that a non-hatched blastocyst resulted from complex perturbations in the regulation of transcription. A-site and B-site hatching embryos, which hatch at the vicinity of the ICM, had a similar rate of implantation but presented different expression profiles. It implies that the multidimensional development of embryos is controlled by ICM, allowing animals to maintain reproduction and continuation of populations. The gene expression profiles in C-site and non-hatched blastocysts clustered together, suggesting that errors made during the blastocyst hatching can completely reverse the developmental fate of the embryo. It may involve the same mechanism for identifying and eliminating abnormalities in the female uterus. Understanding the transcriptional changes from preimplantation to post-implantation embryo development will reveal the implantation mechanism and improve the efficiency of ART.

The study of peri-implantation embryonic development has increased our understanding of embryonic developmental events. Single-cell RNA sequencing of the ovine conceptus and the corresponding endometrium at pre- and peri-implantation stages revealed that an elongated conceptus differentiated into 17 cell types, indicating dramatic cell fate specification (Jia et al., 2023). In the cow, Scatolin et al. defined the cellular composition and gene expression profiles of the embryonic disc, hypoblast, and trophoblast lineages in bovine peri-implantation embryos using single-cell transcriptomes, and compared embryonic peri-implantation lineage programs between bovine and other mammalian species (Scatolin et al., 2024). However, there is much to learn as it is still not possible to predict implantation results by screening an embryo for transfer (Yao et al., 2019). Embryos are usually selected for transfer based on morphological criteria such as the integrity of the blastocyst cavity and the number of inner cell mass/trophectoderm cells (Alegre et al., 2019; Huyen et al., 2018). Even with fresh embryo transfers, the pregnancy rate is less than 54% in humans (Boylan et al., 2020). New technologies combining artificial intelligence with morphokinetic parameters have been developed to screen embryos (Zaninovic and Rosenwaks, 2020), as well as omics-based approaches (Poh et al., 2023). Although these approaches have identified many candidate biomarkers, there is no evidence that they can be used to accurately screen embryos for implantation potential (Aguilera et al., 2024). Here, using key genes involved in blastocyst hatching and hatching outcomes that represent implantation potential, we developed a predictive model for implantation potential for expanding blastocysts. This model, which was weighted using secretory proteins as biomarkers, could provide a non-invasive approach to select embryos for transfer. We are working hard to develop a single embryo detection method that can detect 5 μL culture medium after 2 h embryo culture. Through retrospective study after the delivery of transferred embryos, the embryo culture medium can be detected to validate the effect of this predictive model. With a prospective randomized trial before embryo transfer, embryos with good implantation potential can be selected for transfer to improve the birth rate. As developmental genes are functionally conserved in mammals, this mouse model may be useful for breeding in animals and assisted reproduction in humans.

In conclusion, we analyzed the gene expression profiles of blastocysts during hatching and found changes in transcription patterns that likely determine the hatching phenotype, the hatching process, and hatching outcomes, revealing the molecular changes that prepare blastocyst hatching for implantation. We established a predictive model for implantation success for blastocyst screening. We suggest that transcriptional changes during the development of the preimplantation blastocyst affect its implantation. This study contributes to our understanding of mammalian embryo development during hatching, allowing us to improve our practice in ART.

## Data Availability

The datasets presented in this study can be found in online repositories. The names of the repository/repositories and accession number(s) can be found in the article/Supplementary Material.
