# Supplementary material for: Gene expression changes in blastocyst hatching affect embryo implantation success in mice
Source: Front Cell Dev Biol. 2025 Feb 6;13:1496298. doi: 10.3389/fcell.2025.1496298 (PMC11839805; doi:10.3389/fcell.2025.1496298)
Supplement: Supplementary file 1 [file Table2.docx]

**The primers used in qPCR**

1. C3 F: CCAGCTCCCCATTAGCTCTG

C3 R: GCACTTGCCTCTTTAGGAAGTC

1. IL-1α F: AGGGAGTCAACTCATTGGCG

IL-1α R: CTTCCCGTTGCTTGACGTTG

1. Susd4 F：GGAATGAACCCGAGCAATGGA

Susd4 R：CGCCAGTTGAAACCACAGG

1. Ptgs1 F：ATGAGTCGAAGGAGTCTCTCG

Ptgs1 R：GCACGGATAGTAACAACAGGGA

1. Cd36 F：ATGAATGGTTGAGACCCCGT

Cd36 R：GGAAACCATCCACCAGTTGC

1. Ccl9 F：CCCTCTCCTTCCTCATTCTTACA

Ccl9 R：AGTCTTGAAAGCCCATGTGAAA

1. Cfb F：GAGCGCAACTCCAGTGCTT

Cfb R：GAGGGACATAGGTACTCCAGG

1. Ccl5 F：GCTGCTTTGCCTACCTCTCC

Ccl5 R：TCGAGTGACAAACACGACTGC

1. Lyz2 F：ATGGAATGGCTGGCTACTATGG

Lyz2 R：ACCAGTATCGGCTATTGATCTGA

10、Cyp17a1 F：GCCCAAGTCAAAGACACCTAAT

Cyp17a1 R：GTACCCAGGCGAAGAGAATAGA

11、Podxl F：GCCACCAAAGTGCCACAAC

Podxl F：CGGCATAGATGGAGATTGGGTT

12、Lama1 F：CAGCGCCAATGCTACCTGT

Lama1 F：GGATTCGTACTGTTACCGTCACA

13、Ptk2b F：TGAGCCCTTGAGCCGTGTA

Ptk2b R：AGCTTGAAGTTCTTCCCTGGG

14、Amigo2 F：CTGTGTCTGTTGGTGATCGCA
Amigo2 R：CGGGCACCTTAGATAGGTTTTTG

15、Pramel17 F：CCCACCAGAGGTTATCAGCG
Pramel17 R：TCGCAACACTAAAGTAGAAGTCG

16、Pramel31 F：TGCAGGCAACCTCACTGTAG
Pramel31 R：GCAATCCTTCAGGGGAGGA
